# Complement Inactivation Strategy of *Staphylococcus aureus* Using Decay-Accelerating Factor and the Response of Infected HaCaT Cells

**DOI:** 10.3390/ijms22084015

**Published:** 2021-04-13

**Authors:** Kyoung Ok Jang, Youn Woo Lee, Hangeun Kim, Dae Kyun Chung

**Affiliations:** 1 Graduate School of Biotechnology, Kyung Hee University, Yongin 17104, Korea; cko53@nate.com (K.O.J.); younwoo1093@khu.ac.kr (Y.W.L.); 2 Research and Development Center, Skin Biotechnology Center Inc., Yongin 17104, Korea; 3 Skin Biotechnology Center, Kyung Hee University, Suwon 16229, Korea

**Keywords:** *Staphylococcus aureus*, decay-accelerating factor, internalization, ESAT-6-like protein EsxB, UL16 binding protein 1, tight junction

## Abstract

*Staphylococcus aureus* is a species of Gram-positive staphylococcus. It can cause sinusitis, respiratory infections, skin infections, and food poisoning. Recently, it was discovered that *S. aureus* infects epithelial cells, but the interaction between *S. aureus* and the host is not well known. In this study, we confirmed *S. aureus* to be internalized by HaCaT cells using the ESAT-6-like protein EsxB and amplified within the host over time by escaping host immunity. *S. aureus* increases the expression of decay-accelerating factor (CD55) on the surfaces of host cells, which inhibits the activation of the complement system. This mechanism makes it possible for *S. aureus* to survive in host cells. *S. aureus*, sufficiently amplified within the host, is released through the initiation of cell death. On the other hand, the infected host cells increase their surface expression of UL16 binding protein 1 to inform immune cells that they are infected and try to be eliminated. These host defense systems seem to involve the alteration of tight junctions and the induction of ligand expression to activate immune cells. Taken together, our study elucidates a novel aspect of the mechanisms of infection and immune system evasion for *S. aureus*.

## 1. Introduction

The opportunistic pathogen *Staphylococcus aureus* is a Gram-positive bacterium that causes skin infections (such as boils, carbuncles, cellulitis, and folliculitis). It is the most common cause of community-associated skin and soft tissue infections. *S. aureus* is present in large quantities on the skin and in the nasal cavities of about one in five healthy people; it is not itself a disease. However, it can enter the skin through a wound and cause serious infections in tissues such as the blood and lungs [1]. ESAT-6 secretion system (Ess) extracellular A (EsxA) and Ess extracellular B (EsxB) are virulence factors secreted by *S. aureus* that are known to enable intracellular *S. aureus* infection [2]. The Esx secretion system was first demonstrated in *Mycobacterium tuberculosis*. The Esx-1 secretion system of *M. tuberculosis* affects the secretion of ESAT-6 and CFP-10, which play an important role in the survival of *M. tuberculosis* during infection despite the actions of macrophages and defense systems [3,4]. *S. aureus* has two ESAT-6-like proteins (EsxA and EsxB) that seem to play an important role during infection [5]. However, although the EsxA and EsxB of *S. aureus* are structurally similar to the ESAT-6 and CFP-10 of *M. tuberculosis*, their function is unclear.

The complement system is an important part of innate and adaptive defenses against pathogens. The activation of the complement system occurs through a series of proteolytic cascades, and the final product, the membrane attack complex (MAC), punctures the surface of the pathogen to remove it. In addition, the complement system induces a cytolytic immune response to tissues infected by bacteria, viruses, and parasites [6]. Complement activation occurs through three major biochemical pathways: the classical, lectin, and alternative pathways. In the acute bacterial infection, the serum level of some components is lowered due to activation via either the classical or the alternative pathway. In *S. aureus* bacteremia patients, some patients showed low C3 and C4 levels, suggesting an activation of the complement via the classical pathway. On the other hand, patients having low C3 and normal C4 levels indicate the activation of the alternative pathway [7]. The majority of complement inhibitors secreted from *S. aureus* act on the alternative pathway to block the amplification loop. A few proteins (including the extracellular adherence protein) inhibit the initial cascades that constitute the classical pathway and the lectin pathway [8]. The following complement inhibitors inhibit these pathway activations: membrane cofactor protein (MCP, CD46), decay-accelerating factor (DAF, CD55), and protectin (CD59) [9]. In previous studies, hepatitis C virus up-regulated CD55 expression, entered a host cell, and then infected other cells [10,11]. However, the relationship between *S. aureus* and CD55 is currently unknown.

*S. aureus* is generally considered as an extracellular pathogen, but the internalization of *S. aureus* by keratinocytes has been reported recently and it seems to allow bacteria to evade the host immune system. However, further studies on the interaction between keratinocytes and *S. aureus* are needed. In this study, we identified a mechanism of infection that *S. aureus* uses against HaCaT cells and confirmed that CD55 was used as a mechanism to evade the host immune system. In addition, we confirmed that the UL16 binding protein 1 (ULBP-1) was activated as a defense mechanism of the host cells against the *S. aureus* infection. Studies on the mechanisms of the infection, proliferation, and excretion of *S. aureus* can help to explain the interaction between *S. aureus* and its host.

## 2. Results

### 2.1. EsxB-Mediated Internalization of S. aureus by HaCaT Cells

**Figure 1:**
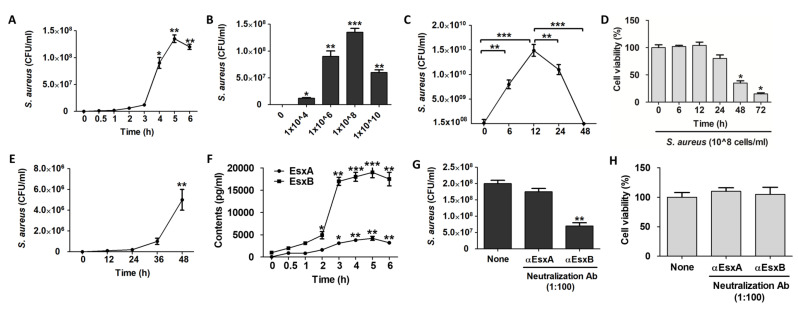
EsxB-mediated internalization of *S. aureus*. (**A**) The colony forming unit (CFU) of intracellular *S. aureus* according to the incubation time of *S. aureus* and HaCaT cells. (**B**) The CFU of intracellular *S. aureus* according to the dose of *S. aureus* administered to HaCaT cells. (C to E) HaCaT cells were cocultured with *S. aureus* for 6 h, washed, and further incubated in a fresh medium with or without gentamycin for the indicated times. Intracellular CFU (**C**), cell viability (**D**), and extracellular CFU from the culture media (**E**) were counted. (**F**) The amount of EsxA and EsxB was examined using indirect ELISA with culture supernatants from HaCaT cells incubated with *S. aureus* for the indicated times. (G to H) HaCaT cells were pretreated with anti-EsxA and anti-EsxB antibodies for 30 min, and then were incubated with *S. aureus* for 6 h. Intracellular CFU were counted (**G**), and a cell viability assay was performed (**H**) after 24 h of further incubation in a fresh medium containing gentamycin. The data displayed are the mean ± SD of three independent experiments. Statistical analysis was conducted using a one-way ANOVA (A to F) or unpaired two-tailed t test (**G**). * *p* < 0.05, ** *p* < 0.01, *** *p* < 0.001 vs. 0 or None.

*S. aureus* has been known as an extracellular pathogen, but recent studies have shown that *S. aureus* can infect human skin epithelial cells [12,13]. In this study, we cocultured HaCaT cells with *S. aureus* for the times indicated in Figure 1A and examined the intracellular colony forming unit (CFU) of *S. aureus*. The number of intracellular *S. aureus* increased significantly beginning 4 h after infection, peaked at 5 h, and then decreased slightly at 6 h (Figure 1A). The decline is believed to result from host cell death. The highest number of intracellular *S. aureus* was about 1.3 × 10^8^ CFU/mL, at 5 h. The number of intracellular *S. aureus* increased dose-dependently up to 1 × 10^8^ CFU, but decreased when cells were treated with 1 × 10^10^ CFU, suggesting that host cell viability was affected by the *S. aureus* population in that culture condition (Figure 1B). The highest number of *S. aureus* within HaCaT cells was about 1.4 × 10^8^ CFU/mL after 5 h of incubation. Based on those two experiments, we determined the best dose and time for an intracellular assay of *S. aureus* in HaCaT cells to be 1 × 10^8^ CFU and 5 h, respectively. Next, we examined how many *S. aureus* can be amplified within HaCaT cells. After 5 h of *S. aureus* infection, HaCaT cells were washed three times with PBS and incubated for the indicated times in fresh, normal culture medium containing gentamicin to remove extracellular *S. aureus*. After 5 h of infection, the number of intracellular *S. aureus* was about 1.5 × 10^8^ CFU/mL, and that number increased significantly for up to 12 h of incubation. The population of *S. aureus* within HaCaT cells began to decrease after 24 h, all the way down to the level exhibited at the 0 time period (Figure 1C). To confirm the correlation between the reduction in intracellular *S. aureus* CFU after 24 h and HaCaT cell death, we performed a toxicity test on HaCaT cells. The WST-1 assay results showed that the HaCaT cells began to die 24 h after infection (Figure 1D). Because cell death was observed after 24 h, we tested whether cell death affected the release of *S. aureus*. We found that the concentration of *S. aureus* in the culture medium increased remarkably beginning 36 h after infection and increased significantly at 48 h (Figure 1E). By that time, *S. aureus* might have been sufficiently amplified and then released by inducing the death of its host cells.

Burts and colleagues have shown that EsxA and EsxB are secreted from *S. aureus* and affect the pathogenesis of *S. aureus* abscesses [5]. Therefore, we examined the level of EsxA and EsxB in the culture supernatants of *S. aureus*-infected HaCaT cells to determine whether EsxA and EsxB were involved in *S. aureus* internalization. We expected to find EsxA and EsxB at the same rate, but we detected more EsxB than EsxA. After 5 h of infection, the EsxB level was about four times higher than that of EsxA (Figure 1F). We found that EsxA and EsxB play different roles in the internalization of *S. aureus* into HaCaT cells. As shown by our neutralization experiments, EsxB appears to play a more important role than EsxA in the internalization of *S. aureus* into host cells (Figure 1G). We used a cytotoxicity test to confirm that the CFU reduction that resulted from neutralizing the EsxB antibody was not caused by the death of the host cells (Figure 1H). Similar to a previous study, our experiments show that EsxB plays an important role in the intracellular internalization of *S. aureus,* but we found that EsxA did not have a significant effect, unlike in previous studies.

### 2.2. CD55 Up-Regulated in S. aureus-Infected HaCaT Cells

**Figure 2:**
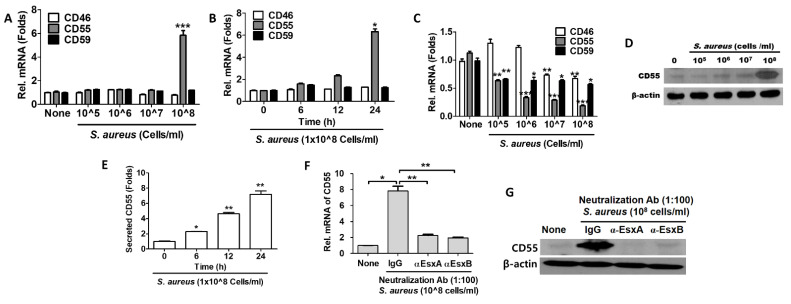
Decay-accelerating factor (CD55) was increased by *S. aureus* infection. HaCaT cells were treated with the indicated doses of *S. aureus* (**A**) for 24 h or with 1 × 10^8^ CFU/mL of *S. aureus* for the indicated times (**B**). In an alternative experiment, HaCaT cells were treated with heat-killed *S. aureus* for 24 h (**C**). The mRNA expression of CIP was examined by real-time PCR and normalized to that of glyceraldehyde 3-phosphate dehydrogenase (GAPDH). (**D**) The CD55 protein level was examined via Western blotting after *S. aureus* treatment. (**E**) Secreted CD55 was examined using indirect ELISA from the culture supernatants of HaCaT cells treated with *S. aureus*. The CD55 mRNA levels (**F**) and protein levels (**G**) of HaCaT cells pretreated with neutralization antibodies and then treated with *S. aureus* were examined by real-time PCR. The data displayed are the mean ± SD of three independent experiments. Statistical analysis was conducted with a one-way ANOVA. * *p* < 0.05, ** *p* < 0.01, *** *p* < 0.001 vs. 0 or None.

Membrane-bound complement regulatory proteins (mCRPs) (such as membrane cofactor protein (MCP/CD46), decay-accelerating factor (DAF/CD55), and the MAC-inhibitory protein (CD59)) inhibit complement responses and affect inflammatory diseases [14,15]. In this study, we examined the role of mCRPs in *S. aureus*-infected skin keratinocytes. First, we used qPCR to examine the mRNA level of mCRPs in HaCaT cells infected with *S. aureus* at the concentrations indicated in Figure 2A. When cells were infected with *S. aureus*, CD55 was increased significantly, but the levels of the other mCRPs were not altered (see Figure 2A). Figure 2B shows that the CD55 mRNA level increased in a time-dependent manner when cells were infected with 1 × 10^8^ CFU/mL of *S. aureus*. To examine whether soluble factors secreted by *S. aureus* affect CD55 induction, we treated HaCaT cells with heat-killed *S. aureus*. Unexpectedly, the mRNA levels of the mCRPs in those cells decreased in a dose-dependent manner (Figure 2C). This result suggests that soluble factors produced by *S. aureus* might regulate the CD55 production of HaCaT cells. However, it is currently unknown why the mCRPs were decreased by heat-killed *S. aureus*. We examined the intracellular protein level of CD55 using Western blotting and observed that CD55 increased dramatically upon infection with 1 × 10^8^ CFU/mL of *S. aureus* (Figure 2D). A dose-dependent increase in soluble CD55 was also observed in the culture supernatants from *S. aureus*-infected HaCaT cells (Figure 2E). As shown in Figure 1, EsxB played an important role in *S. aureus* internalization by HaCaT cells. Therefore, we examined whether EsxB also played a role in CD55 induction during *S. aureus* infection. The mRNA level of CD55 increased in IgG-treated cells, but it was significantly decreased in cells treated with neutralization antibodies (Figure 2F). The protein level of CD55 also did not increase in cells treated with anti-EsxA and anti-EsxB antibodies, indicating that both EsxA and EsxB are involved in CD55 synthesis in *S. aureus*-infected HaCaT cells (Figure 2G). Our results suggest that *S. aureus* secreted EsxA and EsxB in the culture supernatants, which stimulated the cells to produce CD55. The increased CD55 then played a role in inhibiting the complement-mediated immune system. Therefore, it could be used by *S. aureus* to evade host immunity.

### 2.3. S. aureus Inhibited the Complement System

**Figure 3:**
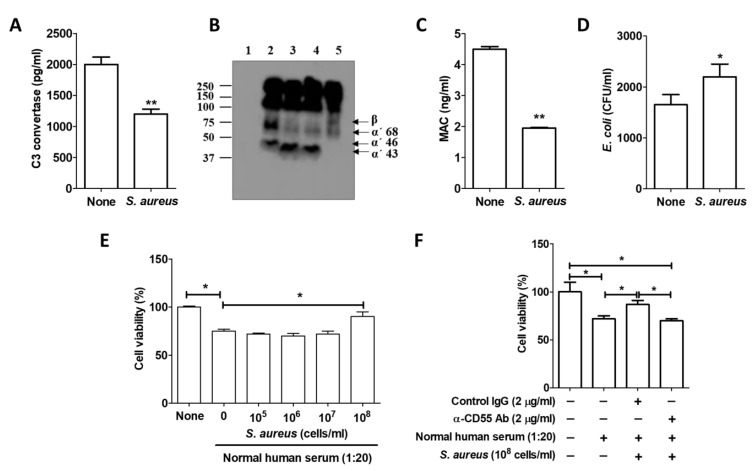
*S. aureus* infection inhibited the complement system. Mice (*n* = 5) were injected with 5 × 10^7^/kg of *S. aureus*, and blood was collected after 24 h. (**A**) The serum C3C level was examined using a commercially available C3C ELISA kit. (**B**) C3 cleavages were examined by Western blotting. Lane 1: HaCaT cell culture media; Lane 2: NHS (1:20 diluted); Lane 3: NHS incubated with *S. aureus*; Lane 4: Coculture media from HaCaT cells and NHS; Lane 5: Coculture media from *S. aureus*-infected HaCaT cells and NHS. (**C**) The serum MAC level was examined using a commercially available MAC ELISA kit. (**D**) A bactericidal assay was performed with normal mouse sera and *S. aureus*-infected mouse sera. (**E**) A complement dependent cytotoxicity (CDC) assay was performed with HaCaT cells and NHS. (**F**) A CDC assay was performed with neutralization antibody–treated HaCaT cells and NHS. The data displayed are the mean ± SD. Statistical analysis was conducted with an unpaired two-tailed t test. * *p* < 0.05, ** *p* < 0.01 vs. None.

MAC, a final product of the complement cascades, induces a cytolytic immune response to remove infected tissue [6]. In these experiments, we examined the alteration of C3, C3C, and MAC activities by *S. aureus* infection. The amount of C3 convertase in the sera of *S. aureus*-infected mice was lower than in normal mouse sera (Figure 3A). C3 cleavages of NHS, which was added to the *S. aureus*-infected HaCaT cells, also diminished (Figure 3B). The amount of MAC in the sera was lowered by *S. aureus* infection (Figure 3C). We used a bactericidal assay to examine the activity of MAC in sera collected from normal and *S. aureus*-infected mice. Figure 3D shows that MAC activity in the sera of *S. aureus*-infected mice was lower than that in normal mouse sera. A complement dependent cytotoxicity (CDC) assay using NHS revealed that MAC activity was inhibited in HaCaT cells treated with 1 × 10^8^ CFU/mL of *S. aureus* (Figure 3E), suggesting that infected HaCaT cells blocked MAC formation on the cell surface by inducing CD55 expression. When HaCaT cells were treated with NHS, cell viability was reduced by about 25%. However, it recovered to about 90% when *S. aureus* was administered. When cells were pretreated with anti-CD55 neutralization antibodies, no recovery of viability occurred with *S. aureus* treatment (Figure 3F). Those results suggest that the CD55 induced by *S. aureus* infection inhibited complement-mediated cytotoxicity in HaCaT cells. As a result, we believe that *S. aureus* can be amplified in skin keratinocytes by avoiding the complement-mediated immune activity of the host.

### 2.4. S. aureus-Induced Cell Death Enables Release from Host Cells

**Figure 4:**
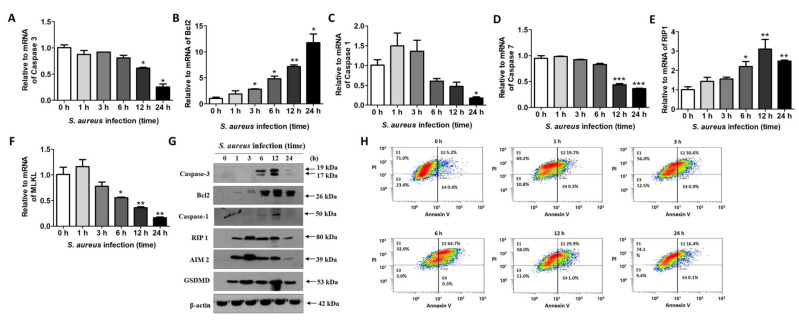
*S. aureus*-mediated cell death in HaCaT cells. HaCaT cells were treated with 1 × 10^8^ CFU/mL of *S. aureus* for the indicated times. Real-time PCR was performed to examine the mRNA levels of caspase-3 (**A**), Bcl2 (**B**), caspase-1 (**C**), caspase-7 (**D**), Rip1 (**E**), and MLKL (**F**). The data displayed are the mean ± SD of three independent experiments. Statistical analysis was conducted with a one-way ANOVA. * *p* < 0.05, ** *p* < 0.01, *** *p* < 0.001 vs. 0 h. (**G**) Protein levels were examined via Western blotting. (**H**) A fluorescence-activated cell sorting (FACS) analysis was performed to examine the type of cell death that occurred in *S. aureus*-infected HaCaT cells.

*S. aureus* that has been sufficiently amplified in host cells is thought to induce cell death to enable its release outside the cell. To examine the kind of cell death triggered by *S. aureus* infection, we first examined the levels of mRNA related to apoptosis, necrosis, and pyroptosis. Real-time PCR results show that caspase-3 mRNA decreased significantly 12 h after infection (Figure 4A). On the other hand, the cleavage forms of caspase-3 increased after 6 h, indicating that the apoptosis pathway was activated by *S. aureus* (Figure 4G). However, Bcl2, an apoptosis inhibitor, increased significantly from 3 h after infection until 24 h after infection (Figure 4B), suggesting that *S. aureus*-induced apoptosis might be blocked by Bcl2. Next, we examined the mRNA variations in caspase-1 and caspase-7, which are involved in pyroptosis. Similar to caspase-3, the mRNA levels of caspases 1 and 7 were decreased by *S. aureus* (Figure 4C,D). At the protein level, caspase-1, AIM2, and gasdermin D (GSDMD) were increased by *S. aureus*, suggesting the occurrence of *S. aureus*-induced pyroptosis, but we are unsure whether the amount of active caspase-1 we found is enough to induce cell death (Figure 4G). We also observed increases in Rip1 at the mRNA (Figure 4E) and protein (Figure 4G) levels, which are related to necrosis. RIP1, RIP3, and MLKL activation are all closely related to necrosis [16]. MLKL was significantly decreased by *S. aureus* (Figure 4F). After infection with *S. aureus*, HaCaT cells were stained with annexin V and PI to examine whether apoptosis occurred. A fluorescence-activated cell sorting (FACS) analysis revealed that the occurrence of necrosis or pyroptosis, but not apoptosis, increased over time in HaCaT cells after *S. aureus* infection (Figure 4H). We do not yet know whether *S. aureus*-induced cell death is necrosis or pyroptosis, but *S. aureus* does not seem to kill host cells by apoptosis.

### 2.5. Host Cells Resist S. aureus Infection Using ULBP-1

**Figure 5:**
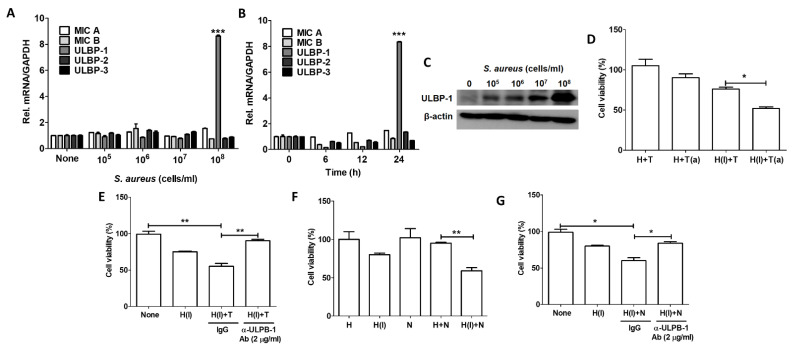
*S. aureus*-infected HaCaT cells produced increased ULBP-1. The mRNA levels of NKG2D ligands were examined by real-time PCR according to the *S. aureus* dose (**A**) and treatment time (**B**). The data displayed are the mean ± SD of three independent experiments. Statistical analysis was conducted with a one-way ANOVA. *** *p* < 0.001 vs. 0 h or None. (**C**) The ULBP-1 protein level was examined via Western blotting. (**D**) A cell viability test was performed after coculturing HaCaT cells and THP-1 cells. (**E**) A cell viability test was performed after coculturing HaCaT cells pretreated with a neutralization antibody and THP-1 cells. (**F**) A cell viability test was performed after coculturing HaCaT cells and NK cells. (**G**) A cell viability test was performed after coculturing HaCaT cells pretreated with neutralization antibody and NK cells. (a) Indicates activated THP-1 cells; (I) indicates cells infected by *S. aureus*. The data displayed are the mean ± SD of two independent experiments. Statistical analysis was conducted with an unpaired two-tailed t test. * *p* < 0.05, ** *p* < 0.01.

NKG2D is a receptor that is increased on activated NK cells. Many NKG2D targeting ligands have been identified, the most interesting of which is a pair of proteins called MHC class I chain-related proteins A and B (MICA and MICB) [17,18]. We thought that damaged cells might increase their expression of MICA/B on their cell surfaces to make them recognizable to NK cells. However, MICA/B were not induced in *S. aureus*-infected keratinocytes. Instead, the mRNA level of ULBP-1 increased by about 9-fold in cells infected by 1 × 10^8^ CFU/mL of *S. aureus* (Figure 5A). The induction of ULBP-1 occurred 24 h after infection, although it tended to decrease 6 and 12 h after infection (Figure 5B). The protein level of ULBP-1 also increased with infection by 1 × 10^8^ CFU/mL of *S. aureus* (Figure 5C). Next, we indirectly confirmed the function of ULBP-1 by using a ULBP-1 neutralizing antibody. The viability of HaCaT cells infected by *S. aureus* was decreased via coculture with THP-1 cells, but no reduction in cell viability was observed when uninfected HaCaT and THP-1 cells were cocultured. Moreover, the viability of infected cells was further reduced when they were incubated with THP-1 cells activated by cytokines (Figure 5D), indicating that THP-1 can eliminate infected cells. The cytotoxicity of THP-1 cells to infected HaCaT cells was reduced by treatment with an anti-ULBP-1 neutralization antibody, suggesting that ULBP-1 plays a role in the interaction between THP-1 cells and HaCaT cells (Figure 5E). Similar results were observed in our experiments with NK cells. *S. aureus*-infected HaCaT cells were eliminated by coculture with NK cells (Figure 5F), but the NK-mediated cytotoxicity was reduced by treatment with an anti-ULBP-1 antibody (Figure 5G). Interestingly, NK cells did not affect uninfected HaCaT cells, suggesting that NK cells eliminated only infected cells using the NKG2D-ULBP-1 interaction.

### 2.6. Host Cells Resist S. aureus Infection by Inducing Tight Junction Proteins

**Figure 6:**
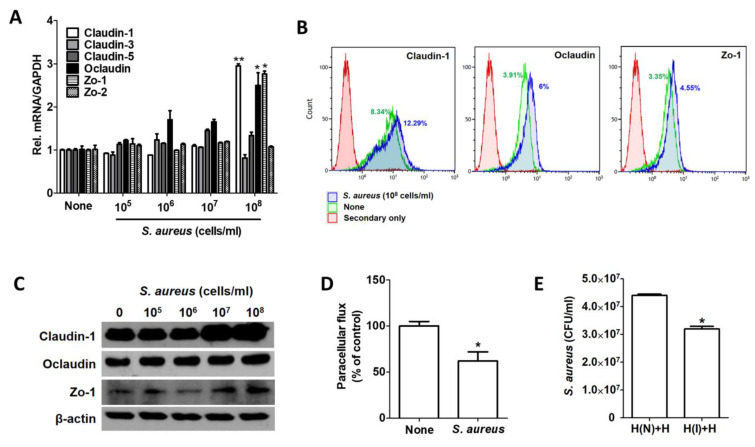
*S. aureus*-infected HaCaT cells increased tight junctions. (**A**) Tight junction–related gene expression was examined by real-time PCR after *S. aureus* infection for 6 h. The tight junction protein level was examined via FACS analysis (**B**) and Western blotting (**C**). (**D**) Paracellular flux was examined by measuring the amount of fluorescein that passed in a Transwell chamber. (**E**) Intracellular *S. aureus* CFU were examined in a Transwell chamber. The data displayed are the mean ± SD. Statistical analysis was conducted with a one-way ANOVA (**A**) or unpaired two-tailed t test (**D**,**E**). * *p* < 0.05, ** *p* < 0.01.

Tight junctions are transmembrane and adapter proteins that seal the most apical portions of intercellular junctions between adjacent epithelial cells. *S. aureus* enhances the integrity of the tight junction barrier by inducing the expression of tight junction molecules from the occludin, tricellulin, and claudin families [19]. We also observed increased caludin-1, occludin, and Zo-1 in HaCaT cells infected by *S. aureus* (Figure 6A). A FACS analysis revealed that the expression of those proteins was increased by 12.29%, 6%, and 4.55%, respectively, in *S. aureus*-infected cells (Figure 6B). The protein most induced by *S. aureus* was thus caludin-1 (Figure 6C). We performed additional experiments with a Transwell chamber to examine the tight junction activity in *S. aureus*-infected HaCaT cells. First, we examined transmission activity by passing fluorescein from the upper chamber to the lower chamber. The fluorescein permeability in *S. aureus*-infected cells was lower than that in uninfected cells (Figure 6D). Similarly, we loaded *S. aureus* into the upper chamber and incubated them with the lower chamber for 24 h. As a control, uninfected cells were cocultured in both the upper and lower chambers. After removing the upper chambers, *S. aureus* was re-administered to the cells grown in both bottom chambers. Intracellular *S. aureus* was decreased in the bottom chamber cocultured with an upper chamber containing *S. aureus* compared with the bottom chamber cocultured with an upper chamber that did not contain *S. aureus* (Figure 6E). These results suggest that host cells infected with *S. aureus* reinforce the tight junctions of neighboring cells as well as their own to inhibit further infection.

## 3. Discussion

*S. aureus* secretes EsxA and EsxB, which seem to be involved in human bacterial pathogenesis [5]. Burts and colleagues have shown that mutant strains of *S. aureus* that failed to secrete EsxA and EsxB displayed a significant reduction in their ability to establish kidney and liver abscesses in infected mice. *S. aureus* EsxA modulates host cell apoptotic pathways, which could provide a haven for amplification. After sufficient amplification, *S. aureus* releases itself from the host cells using EsxA and EsxB [20,21]. In this study, we showed that EsxB (but not EsxA) was involved in *S. aureus* internalization, though exactly how *S. aureus* is internalized into keratinocytes remains unknown. Although more in-depth research is needed, we hypothesize that EsxB increases the efficiency of the interaction between *S. aureus* and its host cells by increasing the expression of host cell receptors such as integrin, TLR2, CD36, and CD14. In our RNA sequencing and qPCR, those receptors increased during *S. aureus* infection, and they did not increase in cells treated with a recombinant EsxB protein. In addition, even in the experiments using EsxB neutralizing antibodies, we found no decrease in the expression of those receptors in the presence of *S. aureus*. Our RNA sequencing analysis revealed that the expression of internalization-related genes (such as ITGN 10, FNDC5, and IGFN1) was more than doubled by the recombinant EsxB protein. However, the overall raw data value was too low, indicating that the reliability was poor (Data not shown). EsxB can regulate host intracellular signaling and thereby control cytokine expression [21]. The exact mechanism by which it accomplishes this is unknown, but EsxB seems to affect susceptibility to *S. aureus* by modulating the intracellular signaling of host cells. *S. aureus* secretes several complement inhibitory molecules such as extracellular fibrinogen-binding protein (Efb), extracellular complement-binding protein (Ecb), staphylococcal complement inhibitor (SCIN) families, and the staphylococcal binder of immunoglobulins (Sbi). These proteins specifically inhibit C3 convertase. For example, to block C3 convertase, SCIN binds to C3c and C3b, and Ecb binds to the C3d domain of C3 [22]. Sbi interacts with Factor H and forms tripartite Sbi:C3: Factor H complexes, which results in the inhibition of the alternative pathway [23]. Unlike *S. aureus*, viruses use CD55 produced from host cells. Hepatitis C viruses and human immunodeficiency viruses (type 1) evade the complement system via the acquisition of CD55 during the budding process from human cells [10,24].

The complement system controls foreign pathogen infections as part of the innate immune system [25]. The complement cascade in the classical pathway is initiated by binding of C1q to the fragment crystallizable (Fc) region of immunoglobulin (Ig) M or IgG of the immune complexes, while spontaneous hydrolysis of C3 activates the alternative pathway. The lectin pathway is initiated by the interaction of certain sugars on the bacterial surface with MBL or ficolin. Both alternative and lectin pathways do not require to recognize antibodies bound to bacterial surface. Although each pathway is initiated by a different mechanism, C3 is commonly involved in those pathways as a key factor [26]. The binding of MBL or C3b to bacterial surface leads to the activation of complement cascades in the lectin and alternative pathways, respectively. Meanwhile, Fc binds to C1q and activates C1s in the classical pathway. In those pathways, C3 convertase converts C3 into C3a and C3b. Cleaved C3b can bind to C3 convertases to form C5 convertase, which cleaves C5 into C5a and C5b. C5b then forms a complex with other components such as C6, C7, C8, and C9 and builds up the MAC on the membrane surface of the bacteria. MAC creates pores on the surface membrane, allowing for the free diffusion of molecules in and out of the cell, and the pathogens are destroyed [27]. As a feedback control system, the complement pathway can be down-regulated by complement regulatory proteins such as CD46, CD55, and C59 [14]. CD46 inhibits complement cascades by acting as a cofactor for the factor-I-mediated cleavage of C3b and C4b [28]. CD55 inhibits the cleavage of C3 and C5 by accelerating the decay of C3 and C5 convertase, respectively [29]. CD59 inhibits MAC formation by preventing C9 polymerization with C5b678 [30]. MAC, a final product of the serial cascades of proteolysis-mediated protein activation, mediates the osmotic lysis of infected cells. The induction of CD55 inhibits the activation of the complement system, which reduces opsonization and MAC formation. *S. aureus* specifically induced CD55, but not CD46 and CD59. Initially, we thought that EsxA or EsxB would drive the increased expression of CD55. However, neither recombinant EsxA nor recombinant EsxB protein affected the expression of CD55 (data not shown). CD55 induction could result from toxins secreted by *S. aureus* or from cell wall components such as lipoteichoic acid and peptidoglycan; more in-depth study is needed. CD55 down-regulates the complement system by inhibiting C3 convertase (C3C) activity. C3C belongs to family of serine proteases that cleave C3 into C3a and C3b. C3a is an anaphylatoxin that increases vascular permeability and promotes the extravasation of phagocytes. C3b is used to form C5 convertase or as an opsonin [31]. As a result of the CD55 induced by *S. aureus* infection, we observed a reduction in the serum C3C level, MAC formation, C3 cleavage, bactericidal activity, and CDC. When *S. aureus* was administered, the complement system was activated, and the infected bacteria were removed. However, in our mouse experiments, the overall activity of the complement system was decreased compared with that in normal mice. This decrease in activity could be due to the CD55 expression induced by *S. aureus*. However, our experiment derived its result from a single injection; different results might appear in conditions of chronic inflammation and infection or with repeated injections of *S. aureus*.

Neutrophils, which are important components of the innate immune system, serve as the primary cellular defense against *S. aureus* infection. Neutrophils recognize bacteria through toll-like receptors (TLR) on the cell surface and then activate signaling to express cytokines, chemokines, and reactive oxygen species. Neutrophils can also kill bacteria through phagocytosis [32]. In this study, we tested the cellular defense system of human keratinocytes against *S. aureus* infection. NKG2D ligands are expressed in damaged, transformed, and pathogen-infected cells to allow NK cells and T cells to recognize and remove them [33]. We expected the expression of NKG2D ligands to increase in *S. aureus*-infected skin cells, and we then expected the infected cells to be eliminated by immune cells. However, we found no significant change in the expression of MICA and MICB, which are the most commonly studied NKG2D ligands. Instead, we confirmed that the mRNA expression of ULBP-1 was significantly increased, and the amount of protein shown in the Western blots was also significantly increased by *S. aureus* infection. These results indicate that skin cells infected with *S. aureus* can be recognized by NK cells or T cells and stably removed. In fact, our in vitro study confirmed that ULBP-1 plays an important role in removing infected skin cells through the action of NK cells and THP-1 cells (Figure 6). An interesting question is why the expression of ULBP-1 increased but the expression of MICA or MICB did not. More research will have to be conducted to answer that question, but our experiments show that the expression pathways of these NKG2D ligands will be different. In fact, ULBP-1 undergoes different transcriptional regulation from other NKG2D ligands in head and neck squamous cell carcinoma cells [34]. In addition, MICA/B, which are expressed in most cells, might have been suppressed by *S. aureus*. MICA/B are expressed more broadly in normal tissues, and their expression occurs primarily within cells, with only a small fraction appearing on the cell surfaces of some epithelial and tumor cells [35]. On the other hand, ULBP-1 is expressed in only a few tissues (https://www.proteinatlas.org/ENSG00000111981-ULBP1/tissue, accessed on 12 April 2021). This might be why the expression of ULBP-1 in skin keratinocytes is not restricted by *S. aureus*. In the end, ULBP-1 could be a major defense mechanism against *S. aureus* infection.

In conclusion, the immune system has developed the complement system to deal with external intruders such as bacteria and viruses. On the other hand, pathogens seem to have evolved to evade that system. *S. aureus* developed the EsxA/EsxB system to facilitate intracellular infection and increased the expression of CD55 by the host to suppress the complement system. We found that CD55 induction prevented infected cells from being eliminated by MAC. After sufficient amplification, *S. aureus* induced cell necrosis or pyroptosis and released from infected cells. Host cells, on the other hand, try to prevent further infection by reinforcing tight junctions with neighboring cells and increasing their cell-surface expression of ULBP-1 to inform surrounding cells that they are infected. Understanding the relationships between hosts and pathogens can help control pathogen infections.

## 4. Material and Methods

### 4.1. Cell Culture

HaCaT cells, a spontaneously immortalized human keratinocyte cell line, were cultured in 5% CO_2_ at 37 °C in a humidified culture incubator in a high-glucose Dulbecco’s Modified Eagle’s Medium (DMEM) supplemented with 10% heat-inactivated fetal bovine serum (FBS) and 1% antibiotics (10,000 units/mL penicillin and 10 mg/mL streptomycin mixture, Welgene, Daejeon, Korea). THP-1 cells, a human promonocytic cell line, were purchased from Korean Cell Line Bank (KCLB 40202) and incubated in RPMI-1640 with 10% heat-inactivated FBS and 1% penicillin and streptomycin mixture. For differentiation, phorbol-12-myristate 13-acetate (PMA, Sigma-Aldrich, St. Louis, MO, USA) was added at a final concentration of 10 ng/mL in RPMI 1640 + 1% FBS for 24 h to prime the THP-1 monocytes into macrophage-like cells. The cells were then washed to remove the PMA, given a 24 h rest, and then allowed to differentiate for 2–3 days. NK 3.3 cells, a human natural killer cell line, were kindly provided by Dr. Ranjit and incubated in RPMI-1640 with 15% heat inactivated FBS, 1% penicillin and streptomycin mixture, and 200 IU/mL of recombinant interleukin-2 (R&D Systems, Minneapolis, MN, USA) at 37 °C in a 5% CO_2_ incubator. The cells were kept at 70–80% confluence and subcultured every 2–3 days.

### 4.2. S. aureus Preparation and Infection

*S. aureus* purchased from the American Type Culture Collection (ATCC 29523) was inoculated into a brain heart infusion medium (BHI, BD Biosciences, San Jose, CA, USA) and incubated for 18 h at 37 °C in aerobic conditions. The next day, the cells were transferred to a fresh BHI medium and cultured to the exponential phase (OD_600_ 1.0). They were then harvested via centrifugation at 8000× *g* rpm for 10 min (1580R, Gyrozen, Korea). The pellet was washed with DPBS 3 times and then resuspended in Dulbecco’s phosphate-buffered saline (DPBS) to the desired concentration. For Heat-killed *S. aureus* preparation, *S. aureus* was cultured under the same conditions and then heat-inactivated at 85 °C for 20 min with 10 s of vortexing every 5 min (ThermoE, BioerTechnology, China; Vortex-Genie 2, Scientific Industries, Bohemia, NY, USA). For all experiments, HaCaT cells were seeded onto 6-well plates or 96-well plates at a density of 1 × 10^4^ to 6 × 10^5^ cells/well in normal growth medium. After 18 h of incubation, the medium was replaced with RPMI without antibiotics. 1 × 10^8^ colony forming units (CFU)/mL of *S. aureus* were added to the cultured cells and incubated for 5 h at 37 °C in a humidified 5% CO_2_ incubator (Sanyo, Tokyo, Japan). After that incubation, the cells were washed three times with DPBS and given a fresh culture medium containing 50 μg/mL of gentamycin and 1% penicillin and streptomycin mixture to remove extracellular *S. aureus*. The cells were further incubated for 24 h and then lysed with hypotonic buffer. The lysed cells were plated on BHI plates and incubated at 37 °C for O/N. The CFU were counted.

### 4.3. Reverse Transcription–Quantitative Polymerase Chain Reaction (RT-qPCR)

To extract total RNA, the cells were washed three times with DPBS and then resuspended in Trizol reagent (Invitrogen, Waltham, MA, USA) according to the manufacturer’s protocol. cDNA was synthesized from total RNA using an iScript cDNA synthesis kit (Bio-Rad, Hercules, CA, USA). To quantify the mRNA expression level, RT-qPCR amplification was conducted using a CFX Connect real-time PCR detection system (Bio-Rad), and the PCR products were detected with SYBR Premix Ex II (TaKaRa, Shiga, Japan). The following sequences of forward and reverse primer pairs were used: GAPDH (5′-AAGGTCGGAGTCAACGGATT-3′ and 5′-GCAGTGAGGGTCTCTCTCCT-3′), CD46 (5′-GTGAGGAGCCACCAACATTT-3′ and 5′-GCGGTCATCTGAGACAGGT-3′), CD55 (5′-CAGCACCACCACAAATTGAC-3′ and 5′-CTGAACTGTTGGTGGGACCT-3′), CD59 (5′-CCGCTTGAGGGAAAATGAG-3′ and 5′-CAGAAATGGAGTCACCAGCA-3′), BCL2 (5′-CTGCACCTGACGCCCTTCACC-3′ and 5′-CACATGACCCCACCGAACTCAAAGA-3′), caspase-1 (5′-TGTGGAAGAGCAGAAAGCGA-3′ and 5′-CCCTGGTGTGGTGTGGTTTA-3′), caspase-3 (5′-AAAGAGGAAGCACCAGAACCC-3′ and 5′-GGGTCAGGAACTTCTGCGAG-3′), caspase-7 (5′-GTGGGAACGATGGCAGATGA-3′ and 5′-GAGGGACGGTACAAACGAGG-3′) MICA (5′-GAAGGCTTGCATTCCCTCCA-3′ and 5′-GGGGCATTGTCCATTCCTCA-3′), MICB (5′-TCAACACCCAGTTGGGACAG-3′ and 5′-GAACCAGTGGACCCAGTAGC-3′), ULBP-1 (5′-TGGCAGATGAGGAGAGTTGT-3′ and 5′-AGAAAGGCACAGTGGTGAGT-3′), ULBP-2 (5′-GAGGTGATTCATCTTCCAGG-3′ and 5′-GCCATTAAGGACCCAGAGCG-3′), ULBP-3 (5′-GGAAGAAGAGGCTGGAACCC-3′ and 5′-TCAGATGCCAGGGAGGATGA-3′), claudin-1 (5′-GAGGTGCCCTACTTTGCTGT-3′ and 5′-TCACACGTAGTCTTTCCCGC-3′), claudin-3 (5′-GTCCGTCCGTCCGTCCG-3′ and 5′-GCCCAGCACGGCCAGC-3′), claudin-5 (5′-GCAGAGGCACCAGAACAG-3′ and 5′-CAGACACAGCACCAGACC-3′, occludin (5′-GCCTTTTGCTTCATCGCTTC-3′ and 5′-AACAATGATTAAAGCAAAAG-3′), Zo-1 (5′-CTGAAGAGGATGAAGAGTAT-3′ and 5′-TGAGAATGGACTGGCTTGG-3′), and Zo-2 (5′-TGGAGGGGATGGATGATGAC-3′ and 5′-CGCCGTCCGTATCTTCAAAG-3′). The expression of mRNA was normalized using glyceraldehyde-3-phosphate dehydrogenase (GAPDH) as a housekeeping gene.

### 4.4. Indirect Enzyme-Linked Immunosorbent Assay (ELISA)

We collected supernatants from *S. aureus*-infected HaCaT cells. An ELISA microplate was coated with the supernatants and left overnight at 4 ˚C. The next day, the supernatants were removed from the microplate, and the plate was washed three times with a washing buffer (1X PBS, 0.05% Tween 20). The microplate was blocked with a blocking buffer (1% bovine serum albumin, 5% sucrose, 0.01% sodium azide) for 2 h at room temperature (RT) and then washed three times. Anti-EsxA, anti-EsxB, and anti-CD55 (Santa Cruz Biotechnology, Dallas, TX, USA) antibodies (1:10 dilution) were added to each well. After incubation for 2 h, the microplate was washed three times, and a horseradish peroxidase (HRP)-conjugated antirabbit antibody (Santa Cruz Biotechnology) was added to each well and incubated for 1 h. After three more washes, the HRP substrate (R & D systems) was added to each well. The reaction was stopped using stop solution (0.16 M sulfuric acid), and the absorbance at 450 nm was read and presented after the subtraction of reagent-control values reacting against BSA-coated negative controls.

### 4.5. Western Blotting

Protein samples were prepared from *S. aureus*-infected HaCaT cells using Laemmli buffer and run with 12% (*w*/*v*) SDS-PAGE in a Glycine/Tris/SDS buffer. The separated proteins were transferred onto a polyvinylidene difluoride membrane for 2 h at 100 V, preincubated in blocking buffer (5% (*w*/*v*) nonfat dried milk in Tris-buffered saline with 0.1% (*v*/*v*) Tween-20 (TBS-T)) for 2 h at RT, and then incubated overnight at 4 °C with primary antibodies (diluted to 1:1000 in TBS-T): anti-CD55, anti-ULBP1, anticlaudin-1, antioccludin, anti-Zo-1 (These were purchased at Santa Cruz Biotechnology), anti-MAP kinase (R&D System), anti-BCL2 (Abcam, Cambridge, MA, USA), anticaspase 1 (Abcam), and anticaspase 7 (Cell Signaling Technology, MA, USA). The membrane was then washed 3 times with TBS-T and reacted for 2 h with HRP-conjugated secondary antibodies (diluted to 1:2000 in TBS-T; Santa Cruz). After three more washes with TBS-T, the protein bands were emitted using an ECL Pico Western blotting reagent (Thermo Fisher Scientific Inc., Waltham, MA, USA) and detected with X-ray film. *β*-actin was used as a loading control.

### 4.6. Flow Cytometry

HaCaT cells infected with *S. aureus* were fixed using 4% formaldehyde at 37 °C for 20 min. After cooling for 5 min, the first antibodies, anti-claudin-1, anti-occludin, and anti-Zo-1 (diluted to 1:200 in PBS; these were purchased at Santa Cruz Biotechnology), were reacted at RT for 1 h. After being washed with 0.5 mL ice-cold PBS, the cells were reacted with anti-rabbit IgG FITC (diluted to 1:1000; Santa Cruz Biotechnology) for 30 min. In a separate study, infected cells were treated with annexin V FITC and propidium iodide (PI) to observe the cell death pattern (diluted to 1:100; Abcam). After the cells were resuspended in 0.5 mL PBS, a flow cytometry analysis was performed at Gyeonggido Business & Science Accelerator (GBSA, Suwon, Korea) using FACSAria II cell sorter (BD).

### 4.7. Complement Dependent Cytotoxicity (CDC) Assay

HaCaT cells infected with *S. aureus* were seeded in 96-well plates and incubated overnight. After being washed with DPBS, the cells were pretreated with an anti-CD55 antibody and a control IgG for 30 min. The cells were then incubated in serum-free DMEM containing normal human serum (NHS, 1:20 dilution) for 6 h. To examine cell viability, dead cells were removed via DPBS washing, and the viability of the HaCaT cells was measured with the Calcein AM cell viability assay system (EMD Millipore, #206700, Burlington, MA, USA). Briefly, 2 µM Calcein AM (final concentration) was added to each well and incubated for 15 min at 37 °C under CO_2_. Fluorescence was examined at 490 nm excitation and 520 nm emission wavelengths.

### 4.8. Bactericidal Assay

Experiments were conducted by modifying a previous method for Gram-negative bacteria [36]. Briefly, *Escherichia coli* DH5α (1 × 10⁴ cells) was reacted with serum isolated from mice (1:50 dilution) for 1 h at 37 °C. After being washed, the *E. coli* cells were serially diluted in PBS, plated with LB medium, and incubated for 16 h at 37 °C. *E. coli* CFU were counted, and differences between the groups were observed.

### 4.9. Mice

5-week-old male Balb/c mice were purchased from Nara Biotech (Seoul, Korea). The mice were cared for in accordance with the guidelines of the animal ethics committee of Kyung Hee University. Approval for the mouse experiments was obtained from the Department of Laboratory Animals, Institutional Animal Care and Use Committee at the global campus of Kyung Hee University (KHU-14-021). A specific pathogen free environment was maintained at a 55% moisture condition, with 16 air changes/h and a cycle of 12 h light and 12 h dark. The mice were divided into two groups (*n* = 5/group): *S. aureus* (5 × 10^7^ CFU/kg) and DPBS injection groups. 100 μL of bacteria were injected subcutaneously. 24 h after that injection, mouse blood was collected from the eyes using a capillary tube. Blood samples were left at RT for 1 h, and then serum samples were separated by centrifuge at 12,000× *g* rpm for 30 min and stored at –80 °C until use. Changes in the activity and quantity of complement factors were measured using commercially available MAC and C3C kits (MyBioSource, San Diego, CA, USA). In addition, mouse blood was used in a bactericidal assay.

### 4.10. Generation of Anti-EsxA and EsxB Polyclonal Antibodies

Recombinant EsxA and EsxB proteins were prepared in *E. coli* BL21(DE3) using the pET28a vector system. Polyclonal antibodies were prepared using rabbits, with recombinant proteins purified through a His-tag column (TaKaRa Korea, Seoul, Korea).

### 4.11. Normal Human Sera Preparation

Whole human blood was purchased from the Blood Center of the Korean Red Cross (Suwon, Korea) and stored at −70 °C until use. To prepare human sera, whole blood was allowed to clot by leaving it undisturbed at RT for 30 min. The clot was removed via centrifugation at 2000× *g* for 10 min in a refrigerated centrifuge. The liquid component was then transferred to a clean polypropylene tube and stored at −20 °C.

### 4.12. Statistical Analysis

All experiments were repeated at least three times. The data shown are representative results or the mean ± SD of triplicate experiments. Statistical analysis of the experimental data was performed using two-tailed Student’s *t* testing, and significant differences between the means of different groups were assessed via one-way analysis of variance (ANOVA) or unpaired two-tailed *t* testing. When the *p* value was lower than 0.05, we deemed the differences to be statistically significant.

## Data Availability

The data presented in this study are available on request from the corresponding authors.
